# Atlanta Society of Medicine

**Published:** 1890-10

**Authors:** 


### ATLANTA SOCIETY OF MEDICINE.

SEPTEMBER 2d, 1890.

Dr. W. P. Nicolson in the chair. In absence of regular secretary, L. P. Kennedy was made secretary *pro tem.*

Applications for membership were read from Drs. J. A. Childs and L. P. Stevens. Drs. Gaston, Avary and Kennedy were appointed to report on above application.

On motion of Dr. D. H. Howell the regular rules of election were suspended, and the society proceeded to elect Drs. W. C. Jarnigan and J. W. Hood members of the society, their names having been presented at a former meeting some months ago.

Dr. Nicolson having been requested by the Society to look about for a more commodious and attractive hall for our sessions, reported that he had secured a good room in the old capitol, and leased the same till September, '91, at $18 per month. On motion of Dr. Howell a committee to be known as the carpet committee, consisting of three members, with the president as chairman *ex officio*, was appointed, said committee to be empowered to act for the society in furnishing a hall. Drs. Howell and Hardon were appointed on this committee. Dr. Elkin moved that a committee of two be appointed to canvass the membership to ascertain what they would do towards fitting up the new hall. Drs. Elkin and Jones were appointed on said committee. Dr. McRae moved that the Society tender its thanks to Dr. Nicolson for his generous efforts in behalf of the Society. Dr. Harris moved that the Society issue cards of invitation to the city physicians requesting them to be present at our next stated meeting. Motion carried and the president was requested to extend the invitations.

There being no further business the Society adjourned.

L. P. KENNEDY, Secretary *pro tem.*

W. P. NICOLSON, President.

SEPTEMBER 16, 1890.

Atlanta Society of Medicine met in new hall on Marietta street, Dr. W. P. Nicolson in the chair.

Minutes of former meeting read and approved.

Dr. Gaston, as chairman of committee on applications, reported favorably on Drs. J. A. Childs and L. P. Stevens. The Society proceeded to ballot on these gentlemen, which resulted in their unanimous election.

Dr. W. F. Westmoreland read a paper on "Tracheotomy for Foreign Bodies in the Air Passage," giving statistics *pro* and *con*, advocating early operation after a diagnosis had been reached. He then gave the history of nine operations in which he had assisted, all resulting favorably. Gave technique of operation as doneby himself, made a free incision, litigating blood vessels as they were severed, after expulsion of foreign body; he did not bring the parts together with two silk ligatures, as was once practiced, leaving them in a bow knot that the wound might be inspected at will, but he closed the wound soon after operation with a deep, intermediate and superficial suture, using cat-gut ligature. He always left a small opening at the lower angle of wound for the escape of serum or pus if there should be any.

Dr. A. W. Calhoun said he had seen a number relieved without an operation, expelling the foreign body in the act of coughing. By using cocaine he had removed two foreign bodies with instruments. He had operated once successfully. Several patients falling under his observation, calling for operation, had been turned over to other surgeons, as he disliked to do the operation. Later in the discussion Dr. Calhoun cited a case of a boy, colored, nine years old, who was brought to him, his father thinking he had something in his throat. Nothing was detected. A number of years after, the boy was taken with a supposed attack of pneumonia, after consultation it was decided that there was pus in the thorax, an incision was made, a drainage tube inserted, and in a short while a cocklebur was discharged through the opening, the boy making a good recovery.

Dr. Earnest reported two cases in which he had assisted, both making a good recovery. He was called to see a patient in consultation, that had been suffering from choking sensation. While they had the patient under observation he had no symptoms, and there was no evidence of a foreign body, but soon after they left the patient died of asphyxia. Reported a fourth case that died ten days after operation from emphysema. Dr. Elkin said he had recently seen a child, aged three years, that had lodged a piece of peanut hull in its throat, followed by paroxyms of coughing, and nearly strangling the child. These paroxyms recur after intervals of two weeks and longer from supposed dislodgement of the body. He thought it was a case for operation, but parents objected. Dr. Elkin thought the anatomy of the parts had some thing to do with the result of the operation. He favored early operation.

Dr. Cooper spoke of the unreliability of statistics. Advocated early operation.

Dr. Nicolson reported progress on the part of committee to furnish the hall.

Dr. Westmoreland moved that a committee be appointed to devise some plan by which the membership may be increased. Drs. Earnest, Westmoreland and Calhoun were appointed on this committee. On motion of Dr. Joye, a decorating committee was appointed, consisting of Drs. Calhoun, Elkin and Olmstead. On motion of Dr. Joye, a janitor was employed at a salary of $1 per month. Society adjourned.

W. P. NICOLSON, President.

L. P. KENNEDY, Secretary *pro tem.*

